# The crystal structures of methyl prop-2-ynoate, dimethyl fumarate and their protonated species

**DOI:** 10.1107/S2053229624011653

**Published:** 2025-01-01

**Authors:** Dirk Hollenwäger, Valentin Bockmair, Andreas J. Kornath

**Affiliations:** a Department Chemie, Ludwig-Maximilians Universität, Butenandtstrasse 5-13 (Haus D), D-81377 München, Germany; J-PARC Center, Japan Atomic Energy Agency, Japan

**Keywords:** crystal structure, superacidic system, ester, protonation, Raman spectroscopy

## Abstract

Methyl prop-2-ynoate (C_4_H_4_O_2_) was investigated in the binary superacidic system HF/*M*F_5_ (*M* = Sb or As) and dimethyl fumarate (C_6_H_8_O_4_) in the superacidic system HF/SbF_5_, as well as HF/BF_3_. The starting materials methyl prop-2-ynoate and dimethyl fumarate were crystallized, the former for the first time. The protonated species of these esters, namely, (1-meth­oxy­prop-2-yn-1-yl­idene)oxidanium hexa­fluoro­arsenate, C_4_H_5_O_2_^+^ AsF_6_^−^, 1,4-dimeth­oxy-4-oxo­but-2-en-1-yl­idene]oxidanium tetra­fluoro­borate bis(hy­dro­gen fluoride), C_6_H_9_O_4_^+^ BF_4_^−^ 2HF, and hemi{[1,4-dimeth­oxy-4-oxidaniumylidenebut-2-en-1-yl­idene]oxi­dan­ium} undeca­fluoro­diantimonate, 0.5C_6_H_10_O_4_^2+^ Sb_2_F_11_^−^, were characterized by single-crystal X-ray diffraction and Raman spectroscopy. The protonated species were recrystallized from anhydrous hy­dro­gen fluoride. In the solid state of the monoprotonated species of methyl prop-2-ynoate and the diprotonated species of dimethyl fumarate, strong intra­molecular O—H⋯F hy­dro­gen bonds build a three-dimensional network. The monoprotonated species of dimethyl fumarate builds chains by strong O—H⋯O hy­dro­gen bonds between the cations.

## Abstract 2

Methyl prop-2-ynoate and dimethyl fumarate have been crystallized, the former for the first time. The protonated species of both were isolated for the first time and identified by single-crystal X-ray diffraction and Raman spectroscopy.

## Introduction

Protonated esters have occur in two conformations, namely, *syn*–*anti* and *syn*–*syn* (Hogeveen, 1967 ▸; Olah *et al.*, 1967 ▸, 2009 ▸). The *syn*–*anti* conformation is more stable and is therefore consistent with the protonation of carb­oxy­lic acids (Olah *et al.*, 2009 ▸; Hollenwäger *et al.*, 2024*b* ▸; Hogeveen, 1968 ▸). The two conformers were observed in solution by NMR spectroscopy (Olah *et al.*, 2009 ▸). It has not yet been possible to crystallize the *syn*–*syn* conformer of protonated esters in the solid state; the example of prop-2-ynoic acid (propiolic acid) shows that this could be achieved with a H/D exchange and solid-state effects through a larger anion (Hollenwäger *et al.*, 2024*b* ▸). In magic acid (FSO_3_H/SbF_5_), the esters show the unimolecular cleavage of methanol by warming to 20 °C (Olah *et al.*, 2009 ▸). An exception was observed with glycine methyl ester, which is still stable in magic acid even at 93 °C (Hollenwäger, *et al.*, 2024*a* ▸).

The isolation of protonated esters enables the characterization of an important inter­mediate that is present in solution in every acid-catalyzed esterification process. The selected esters also offer the possibility of further functionalization steps due to the double and triple bonds present. This prompted us to investigate methyl prop-2-ynoate and dimethyl fumarate in the binary superacidic media HF/*M*F_5_.

## Experimental

### Synthesis and crystallization

#### [C_4_H_5_O_4_][*M*F_6_] (*M* = Sb or As)

The Lewis acids (SbF_5_: 433 mg, 2 mmol; AsF_5_: 340 mg, 2 mmol) were each condensed into a fluorine-passivated FEP reactor. Anhydrous hy­dro­gen fluoride (0.5 l) was added as reactant and solvent at −196 °C. The mixture was homogenized at room tem­per­a­ture. Methyl prop-2-ynoate (83.6 µl, 84.1 mg, 1.0 mmol) was added at −196 °C under nitro­gen. The mixture was allowed to warm to room tem­per­a­ture. The solvent was removed overnight at −78 °C. The protonated species **II** and **III** were obtained as white solids (Scheme 1).

#### [C_6_H_9_O_4_][*M*F_*y*_] (*M* = Sb or B; *y* = 6 or 4)

The Lewis acids (SbF_5_: 216 mg, 1 mmol; BF_3_: 67 mg, 1 mmol) were each condensed into a fluorine-passivated FEP reactor. Anhydrous hy­dro­gen fluoride (0.5 l) was added as reactant and solvent at −196 °C. The mixture was homogenized at room tem­per­a­ture. Dimethyl fumarate (144 mg, 0.5 mmol) was added at −196 °C under nitro­gen. The mixture was allowed to warm to room tem­per­a­ture. The solvent was removed overnight at −78 °C. The protonated species **V** and **VI** were obtained as white solids (Scheme 1). A clean Raman spectrum of monoprotonated species **VI** could not be obtained because it contains impurities of either **IV** or **VII**.
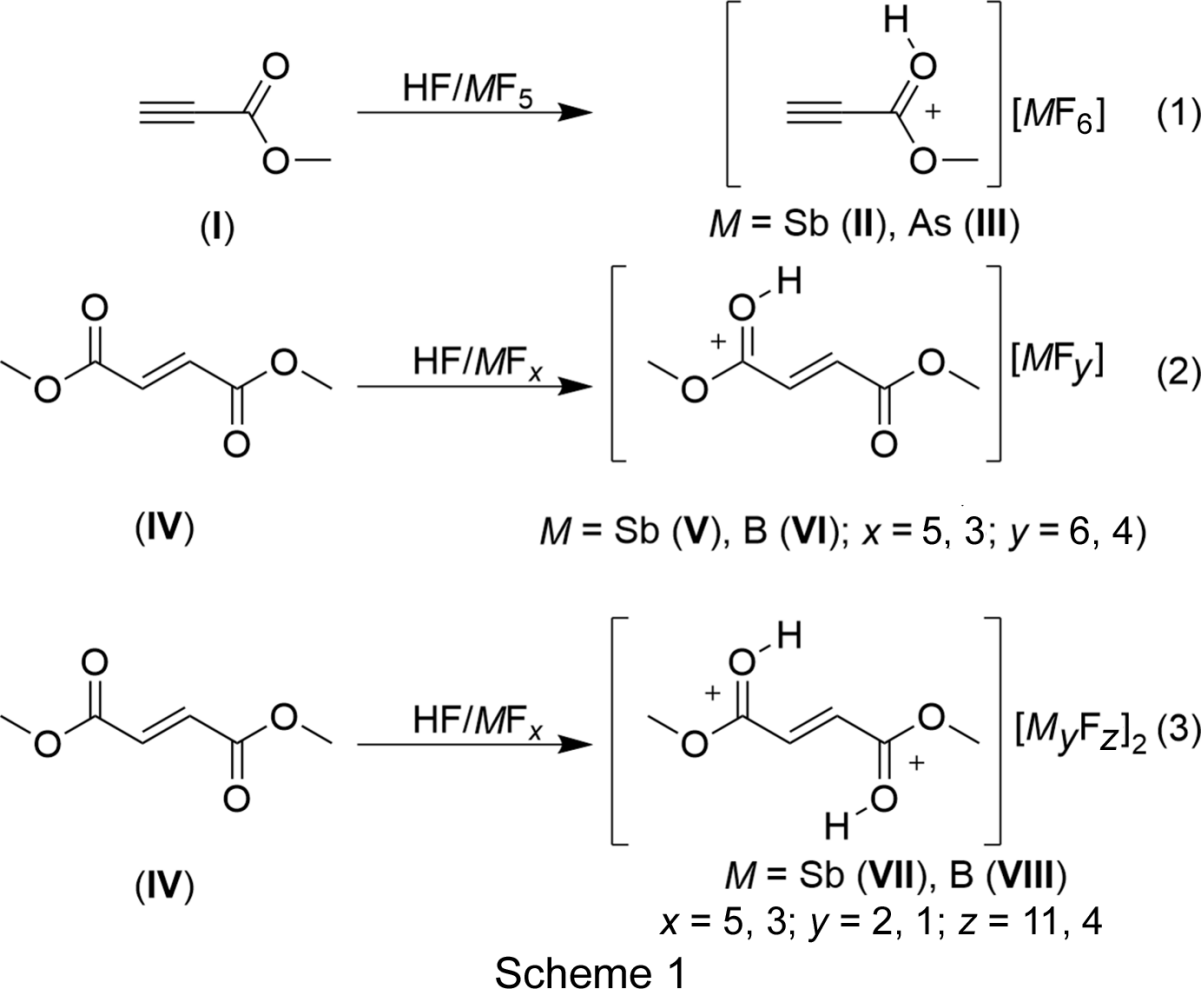


#### [C_6_H_10_O_4_][Sb_2_F_11_]_2_ and [C_6_H_10_O_4_][BF_4_]_2_

The Lewis acids (SbF_5_: 216 mg, 1 mmol; BF_3_: 67 mg, 1 mmol) were each condensed into a fluorine-passivated FEP reactor. Anhydrous hy­dro­gen fluoride (0.5 l) was added as reactant and solvent at −196 °C. The mixture was homogenized at room tem­per­a­ture. Dimethyl fumarate (48 mg, 0.33 mmol) was added at −196 °C under nitro­gen. The mix­ture was allowed to warm to room tem­per­a­ture. The solvent was removed overnight at −78 °C. The protonated species **VII** and **VIII** were obtained as white solids.

### Single-crystal X-ray diffraction and Raman spectroscopic analysis

Com­pounds **I**, **III**, **VI** and **VII** were characterized by single-crystal X-ray diffraction. Com­plete data and devices for the X-ray measurements are listed in the CIF in the supporting information. Low-tem­per­a­ture Raman spectroscopic analysis was performed for **I**–**VIII** using a Bruker MultiRAM FT–Raman spectrometer with Nd:YAG laser excitation (λ = 1064 cm^−1^) under vacuum. For the measurements, the synthesized com­pound was transferred to a cooled glass cell.

### Refinement

**Figure 1:**
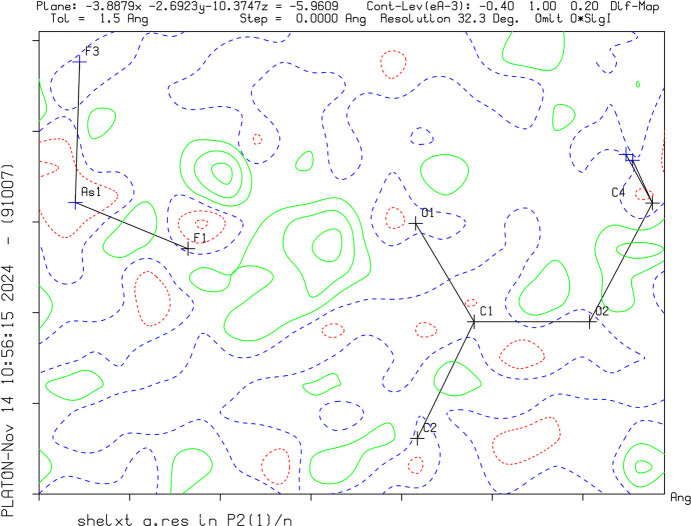
A difference Fourier map of **III** without the H atom between O1 and F1. The green solid lines and red dotted lines show positive and negative density distribution, respectively.

Crystal data, data collection and structure refinement details are summarized in Table 1 ▸. The successful protonation of the target mol­ecules was confirmed by the charge of the asymmetric unit, as well as the interatomic distances. C—O distances have become nearly equal after protonation, as the charge can formally be localized on the C atom resulting in the loss of double-bond character. The positions of the H atoms were identified by Q-peaks on the difference Fourier map and by evaluation of the contacts (Figs. 1 ▸–3 ▸ ▸). Methyl, methylene and acetylenic H atoms were refined under restrictions and the proton positions were modulated.

## Results and discussion

### Single-crystal X-ray diffraction

#### Crystal structure of methyl prop-2-ynoate (I)

**Figure 4:**
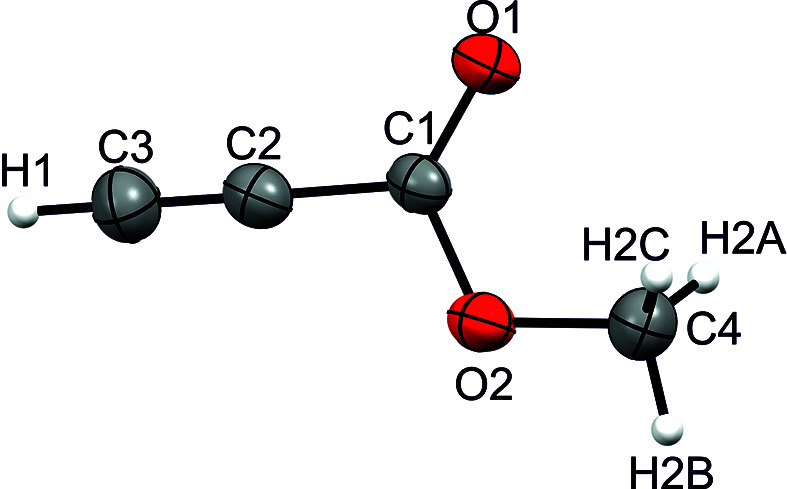
The asymmetric unit of **I**, with displacement ellipsoids drawn at the 50% probability level.

Com­pound **I** crystallizes in the monoclinic space group *P*2_1_/*n* with one formula unit per unit cell. Fig. 4 ▸ displays the asymmetric unit. The C1—C2 bond length [1.4466 (15) Å] is significantly elongated com­pared to an average C*sp*^1^—C*sp*^2^ hybridized bond (1.427 Å) determined by X-ray diffraction (Allen *et al.*, 1987 ▸). The C2≡C3 triple bond [1.1780 (16) Å] is in the same range as average terminal C≡C bonds (1.181 Å; Allen *et al.*, 1987 ▸). The C1=O1 bond [1.1972 (14) Å] is in the same range as other C=O bonds (1.196 Å) in esters (Allen *et al.*, 1987 ▸). The C1—O2 bond [1.3195 (12) Å] is significantly shortened com­pared to other esters (1.337 Å; Allen *et al.*, 1987 ▸). The C4—O2 bond [1.4463 (13) Å] is significantly elongated com­pared to average CH_3_—O bonds in esters (1.418 Å; Allen *et al.*, 1987 ▸).

**Figure 5:**
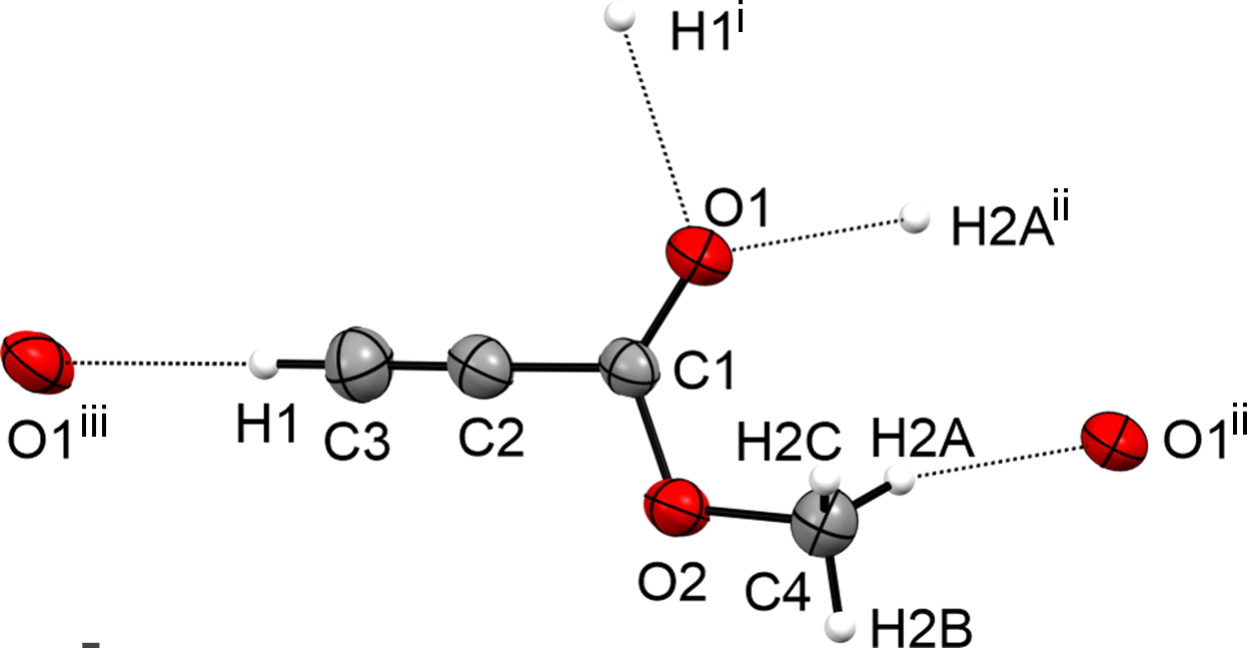
Hy­dro­gen bonds in the crystal structure of **I**, with displacement ellipsoids drawn at the 50% probability level. [Symmetry codes: (i) *x* − 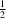, −*y* + 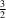, *z* + 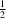; (ii) −*x* + 1, −*y* + 1, −*z* + 2; (iii) *x* + 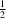, −*y* + 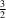, *z* − 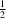.]

The crystal structure of **I** displays a layered structure built of weak C3—H1⋯O1 hy­dro­gen bonds, according to the classification of Jeffrey (1997 ▸). The layered structure is connected *via* weak C4—H2*A*⋯O1 hy­dro­gen bonds into a three-dimensional network (Fig. 5 ▸), according to the classification of Jeffrey (1997 ▸).

#### Crystal structure of (1-meth­oxy­prop-2-yn-1-yl­idene)oxidanium hexa­fluoro­arsenate (III)

**Figure 6:**
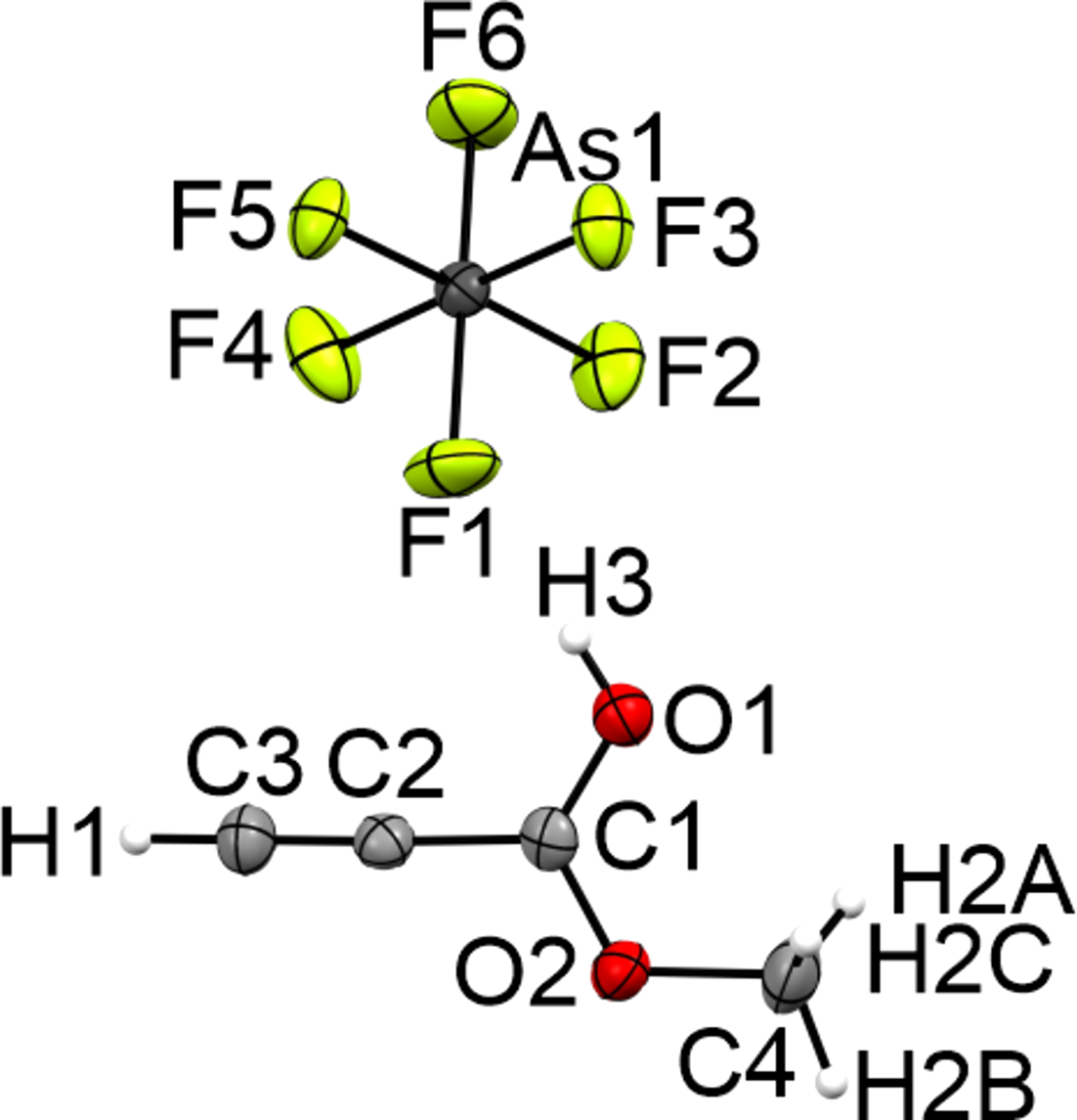
The asymmetric unit of **III**, with displacement ellipsoids drawn at the 50% probability level.

Salt **III** crystallizes in the monoclinic space group *P*2_1_/*n* with four formula units per unit cell. Fig. 6 ▸ displays the asymmetric unit of **III**. The C1—O1 bond [1.261 (4) Å] is significantly elongated by 0.064 Å due to the protonation com­pared to **I**. The C1—O2 bond [1.270 (3) Å] is significantly shortened by 0.049 Å com­pared to the starting material **I**. Due to the protonation, the C4—O2 bond [1.484 (4) °] is elongated by 0.038 Å com­pared to the neutral com­pound **I**. The C2≡C3 triple bond is not significantly influenced by the protonation.

**Figure 7:**
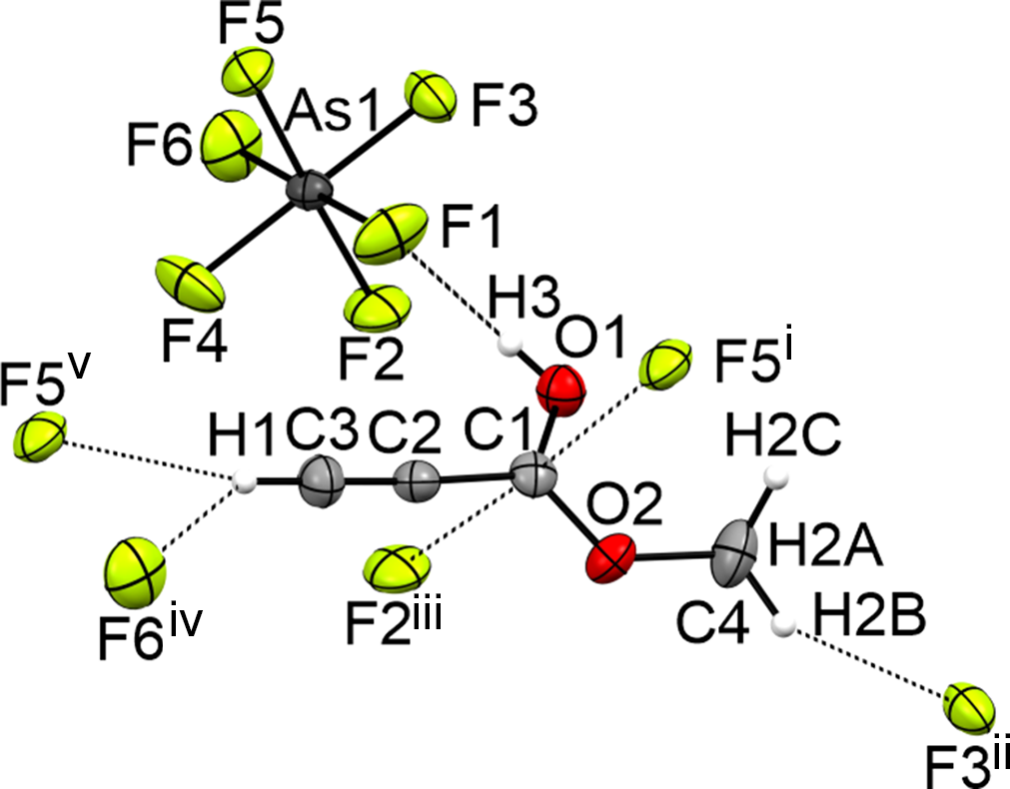
The intra­molecular inter­actions in the crystal structure of **III**, with displacement ellipsoids drawn at the 50% probability level. [Symmetry codes: (i) −*x* + 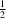, *y* − 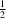, −*z* + 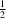; (ii) −*x*, −*y* + 1, −*z* + 1; (iii) −*x* + 1, −*y* + 1, −*z* + 1; (iv) *x*, *y* − 1, *z*; (v) −*x* + 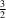, *y* − 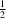, −*z* + 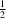.]

The three-dimensional network of **III** (Fig. 7 ▸) is built by a strong O1—H3⋯F1 and three weak C3—H1⋯F5, C3—H1⋯F6 and C4—H2*B*⋯F3 hy­dro­gen bonds, according to the classification of Jeffrey (1997 ▸). Additionally, the crystal structure forms two inter­atomic contacts (C1⋯F2 and C1⋯F5) which are 8% shorter than the sum of the van der Waals radii.

#### Crystal structure of dimethyl (*E*)-but-2-enedioate (IV)

**Figure 8:**
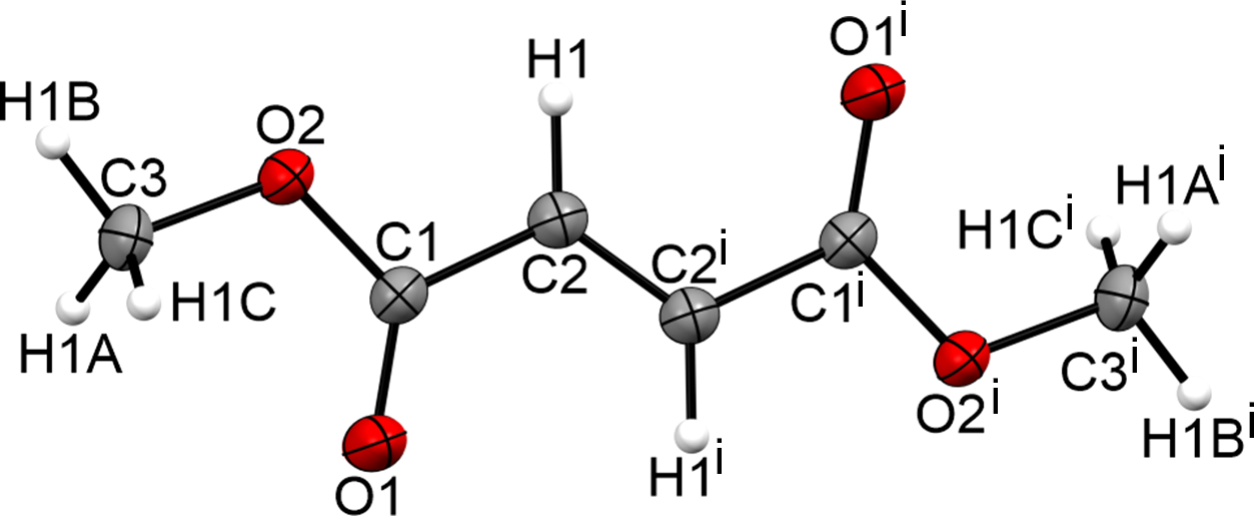
The formula unit of **IV**, with displacement ellipsoids drawn at the 50% probability level. [Symmetry code: (i) −*x* + 1, −*y* + 2, −*z* + 1.]

**Figure 9:**
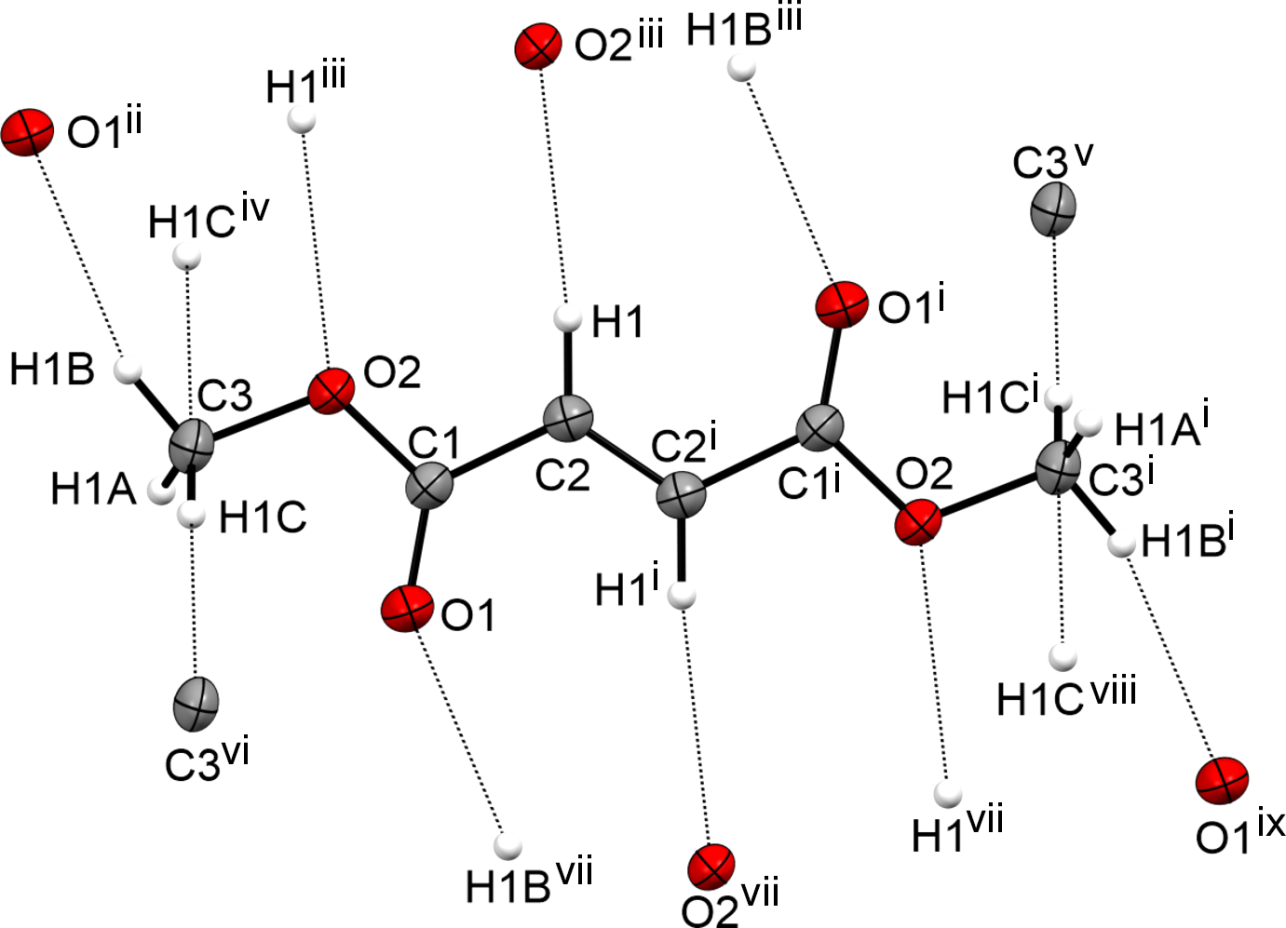
The intra­molecular inter­actions in the crystal structure of **IV**, with displacement ellipsoids drawn at the 50% probability level. [Symmetry codes: (i) −*x* + 1, −*y* + 2, −*z* + 1; (ii) *x* − 1, *y* − 1, *z*; (iii) −*x*, −*y* + 1, −*z* + 1; (iv) *x* − 1, *y*, *z*; (v) −*x*, −*y* + 2, −*z* + 1; (vi) *x* + 1, *y*, *z*; (vii) *x* + 1, *y* + 1, *z*; (viii) −*x* + 2, −*y* + 2, −*z* + 1; (ix) −*x* + 2, −*y* + 3, −*z* + 1.]

The determined crystal structure is in the same range as that reported by Kooijman *et al.* (2004 ▸). The formula unit is shown in Fig. 8 ▸ and the crystal structure exhibits the same three-dimensional network (Fig. 9 ▸).

#### Crystal structure of 1,4-dimeth­oxy-4-oxobut-2-en-1-yl­idene]oxidanium tetra­fluoro­borate–hy­dro­gen fluoride (1/2) (VI)

**Figure 10:**
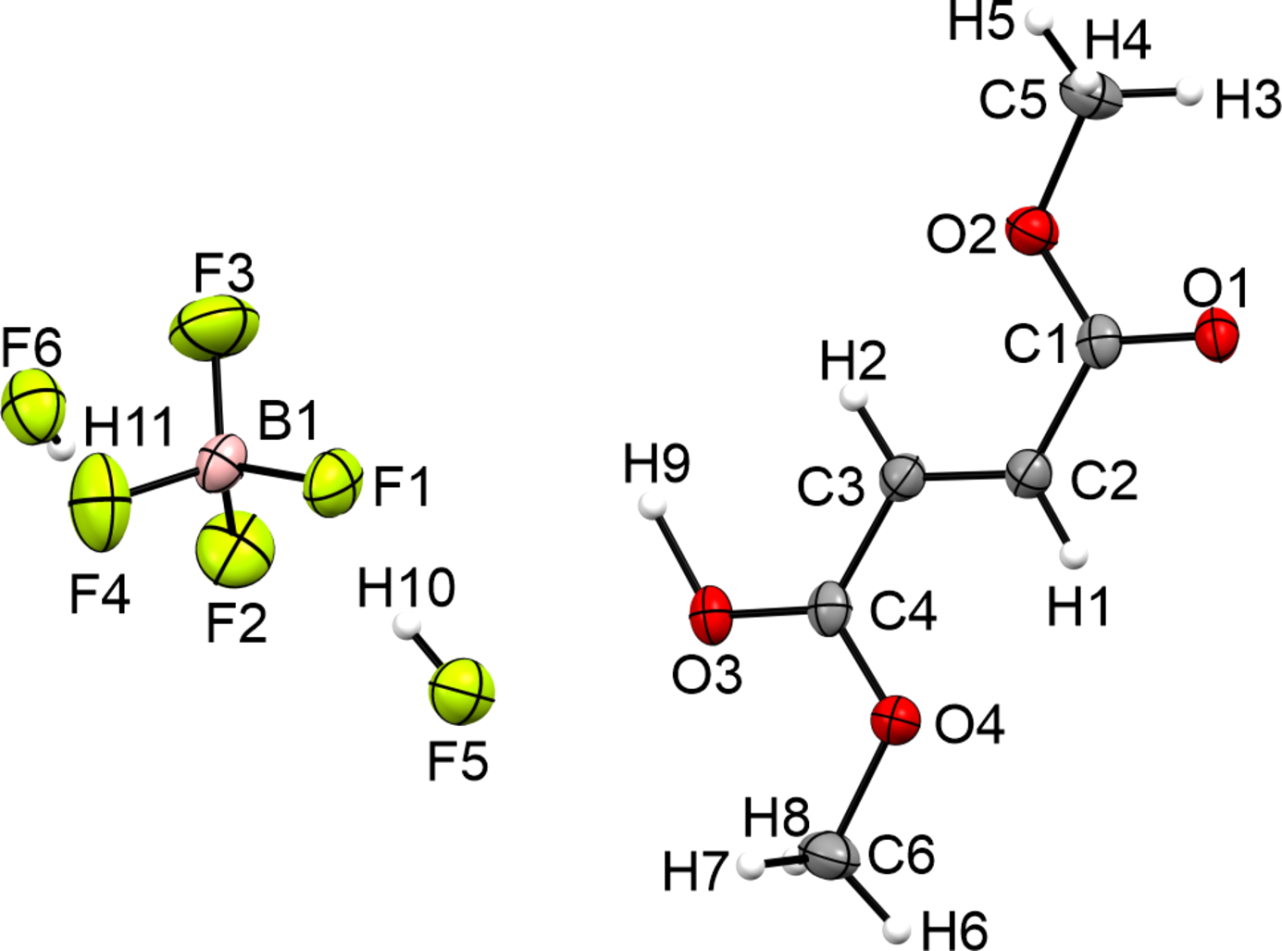
The asymmetric unit of **VI**, with displacement ellipsoids drawn at the 50% probability level.

The crystal structure of the monoprotonated species **VI** of dimethyl fumarate crystallizes in the ortho­rhom­bic space group *Pbca* with eight formula units per unit cell. Fig. 10 ▸ displays the asymmetric unit. Similar to fumaric acid and acetyl­enedi­carb­oxy­lic acid, the monoprotonated type forms extended chains of cations that are connected *via* strong O3—H9⋯O1 hy­dro­gen bonds (Jessen & Kornath, 2022 ▸; Bayer *et al.*, 2020 ▸; Jeffrey, 1997 ▸). Due to the protonation, the C1—O1 [1.248 (3) Å] and C4—O3 [1.247 (3) Å] bonds are significantly elongated com­pared to the neutral com­pound [1.205 (2) Å]. The C1—O2 and C4—O4 bonds [both 1.289 (3) Å] are shortened by 0.052 Å com­pared to the starting material **IV**. The CH_3_—O and C2=C3 bonds are not significantly influenced by the monoprotonation.

**Figure 11:**
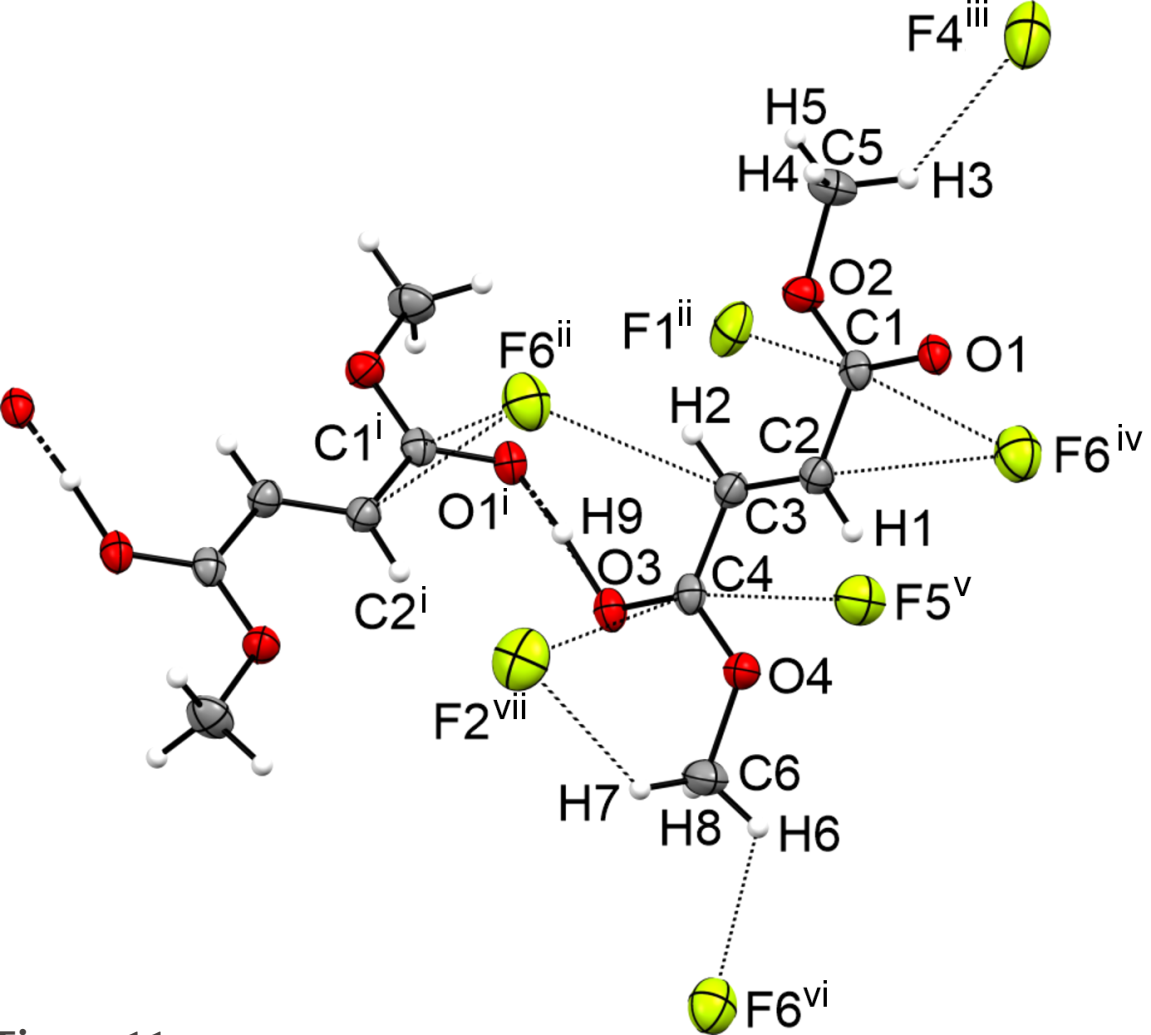
The intra­molecular inter­actions in the crystal structure of **VI**, with displacement ellipsoids drawn at the 50% probability level. [Symmetry codes: (i) −*x* + 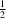, *y* − 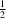, *z*; (ii) −*x* + 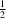, *y* + 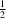, *z*; (iii) *x* − 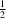, *y* + 1, −*z* + 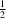; (iv) *x*, *y* + 1, *z*; (v) −*x* + 1, −*y* + 1, −*z* + 1; (vi) *x*, −*y* + 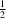, *z* + 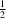; (vii) *x* − 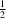, −*y* + 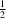, −*z* + 1.]

Besides the strong hy­dro­gen bonding between the cations, the crystal structure forms a medium–strong C6—H6⋯F6 hy­dro­gen bond [3.198 (3) Å] and two weak C5—H3⋯F4 [3.242 (3) Å] and C6—H7⋯F2 [3.261 (3) Å] hy­dro­gen bonds. Furthermore, the crystal structure forms six inter­atomic C⋯F contacts, *i.e.* C1⋯F1 [2.900 (3) Å], C1⋯F6 [2.943 (3) Å], C2⋯F6 [2.987 (3) Å], C3⋯F6 [3.048 (3) Å], C4⋯F2 [3.020 (3) Å] and C4⋯F5 [2.974 (3) Å], which are shorter than the sum of the van der Waals radii (3.17 Å). The inter­actions in the crystal structure of **VI** are shown in Fig. 11 ▸.

#### Crystal structure of hemi{[1,4-dimeth­oxy-4-oxidan­iumylidenebut-2-en-1-yl­idene]oxidanium} undeca­fluoro­diarsenate (VII)

**Figure 12:**
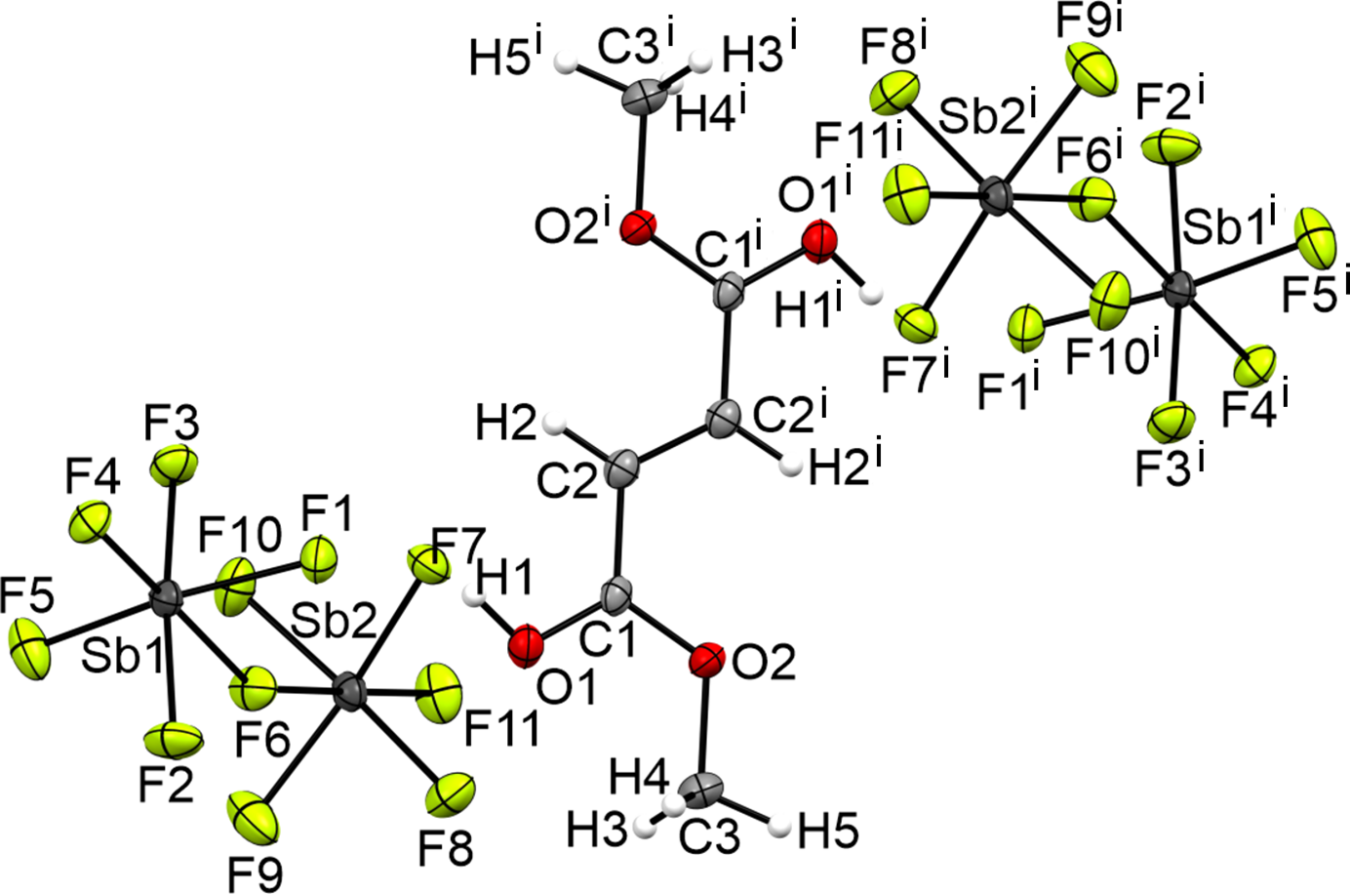
The formula unit of **VIII**, with displacement ellipsoids drawn at the 50% probability level. [Symmetry code: (i) −*x* + 1, −*y* + 1, −*z* + 1.]

The crystal structure of the diprotonated species **VII** of dimethyl fumarate crystallizes in the ortho­rhom­bic space group *Pbca* with eight formula units per unit cell. Fig. 12 ▸ displays the formula unit. The crystal structure of the diprotonated species has a C1—O1 bond [1.277 (5) Å] significantly elongated by 0.072 Å com­pared to the starting material [1.205 (2) Å]. The C1—O2 bond [1.281 (5) Å] is shortened by 0.060 Å com­pared to the neutral com­pound [1.341 (1) Å]. The C3—O2 bond [1.489 (6) Å] is elongated by 0.034 Å com­pared to **IV** [1.455 (2) Å].

**Figure 13:**
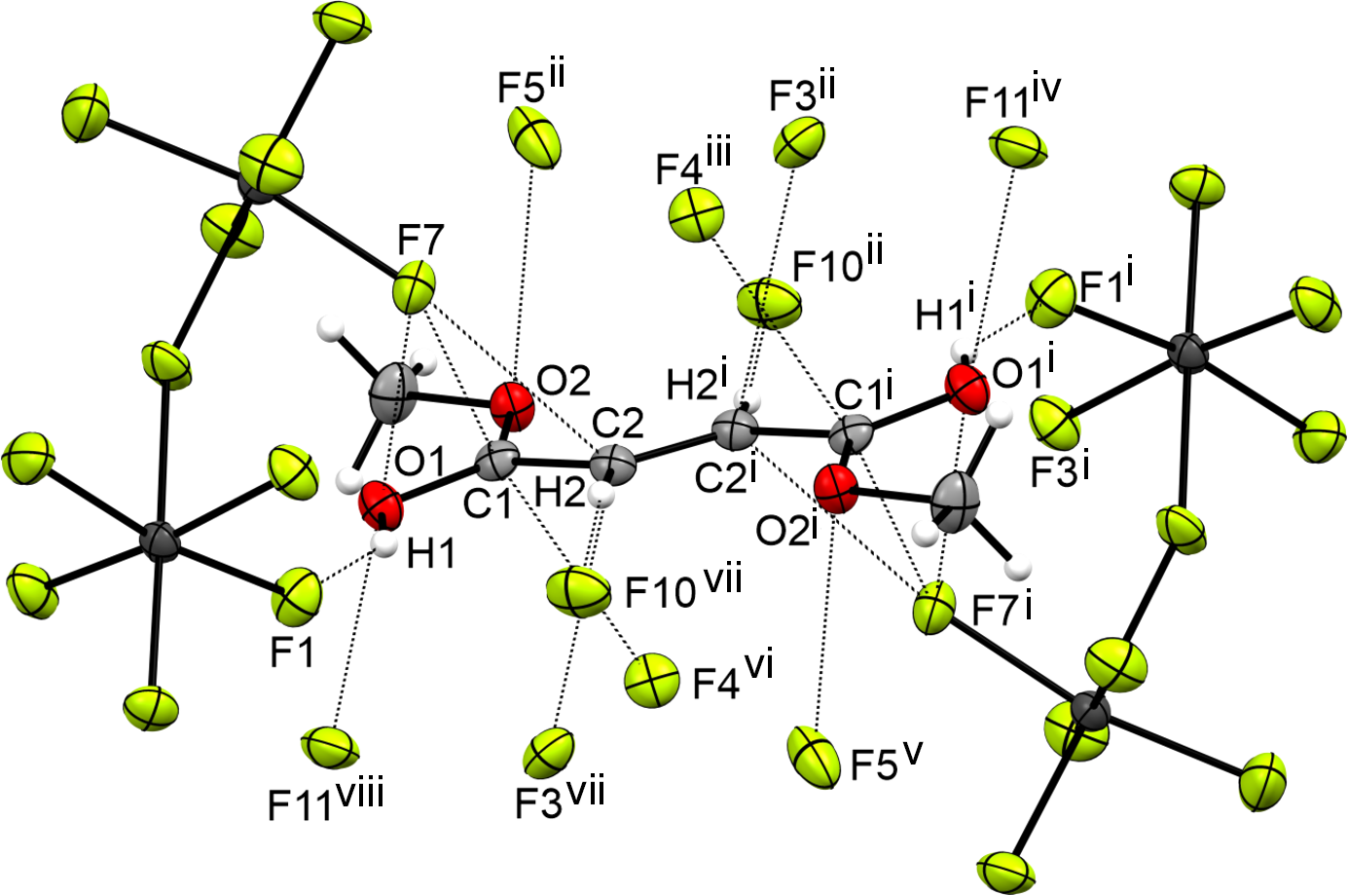
The intra­molecular inter­actions in the crystal structure of **VIII**, with displacement ellipsoids drawn at the 50% probability level. [Symmetry codes: (i) −*x* + 1, −*y* + 1, −*z* + 1; (ii) −*x* + 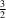, *y* + 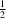, *z*; (iii) *x* + 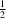, −*y* + 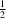, −*z* + 1; (iv) −*x* + 2, −*y* + 1, −*z* + 1; (v) *x* − 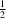, −*y* + 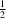, −*z* + 1; (vi) −*x* + 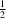, *y* + 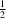, *z*; (vii) *x* − 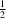, −*y* + 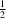, −*z* + 1; (viii) *x* − 1, *y*, *z*.]

The three-dimensional network is formed by a strong O1—H1⋯F1 hy­dro­gen bond and seven inter­atomic inter­actions (Jeffrey, 1997 ▸). The inter­atomic inter­actions are C1⋯F4 [3.061 (4) Å], C1⋯F7 [3.001 (4) Å], C2⋯F3 [2.958 (5) Å], C2⋯F7 [3.008 (5) Å], C3⋯F2 [3.050 (6) Å], O1⋯F7 [2.985 (4) Å] and O1⋯F11 [2.864 (4) Å]. Fig. 13 ▸ displays the inter­atomic distances in the crystal structure of **VII**.

### Raman spectroscopy

#### Raman spectra of I, II and III

**Figure 14:**
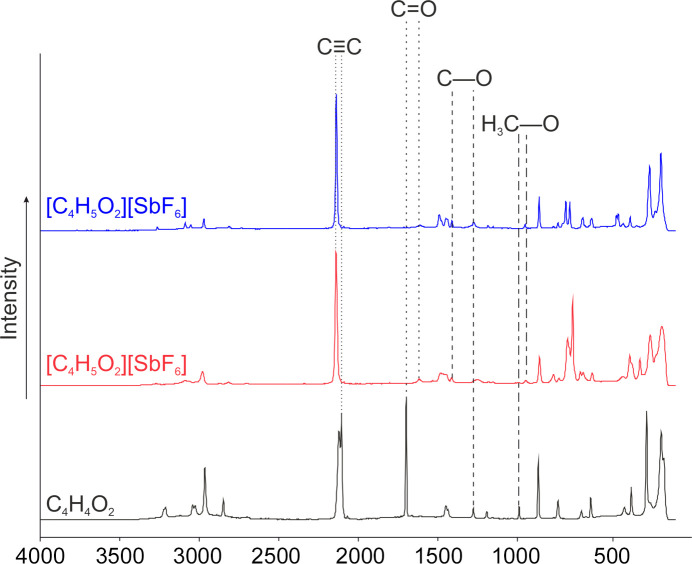
The low-tem­per­a­ture Raman spectra of **I** (black), **II** (red) and **III** (blue).

Fig. 14 ▸ displays the low-tem­per­a­ture Raman spectra of **I**, **II** and **III**. The first evidence for succesful protonation is the significantly red-shifted C=O oscillation from 1700 cm^−1^ in the starting material **I** to 1617 (in **II**) and 1615 cm^−1^ (in **III**). Due to the protonation, the C—O oscillation is blue-shifted in the Raman spectra to 1414 (in **II**) and 1413 cm^−1^ (in **III**) com­pared to the starting material (1278 cm^−1^). The H_3_C—O oscillation is red-shifted by 42 cm^−1^ to 948 (in **II**) and 951 cm^−1^ (in **III**) com­pared to **I** (990 cm^−1^). The oscillation of the triple bond is only slightly affected from 2107 cm^−1^ to 2141 (in **II**) and 2140 cm^−1^ (in **III**).

#### Raman spectra of IV, V, VII and VIII

**Figure 15:**
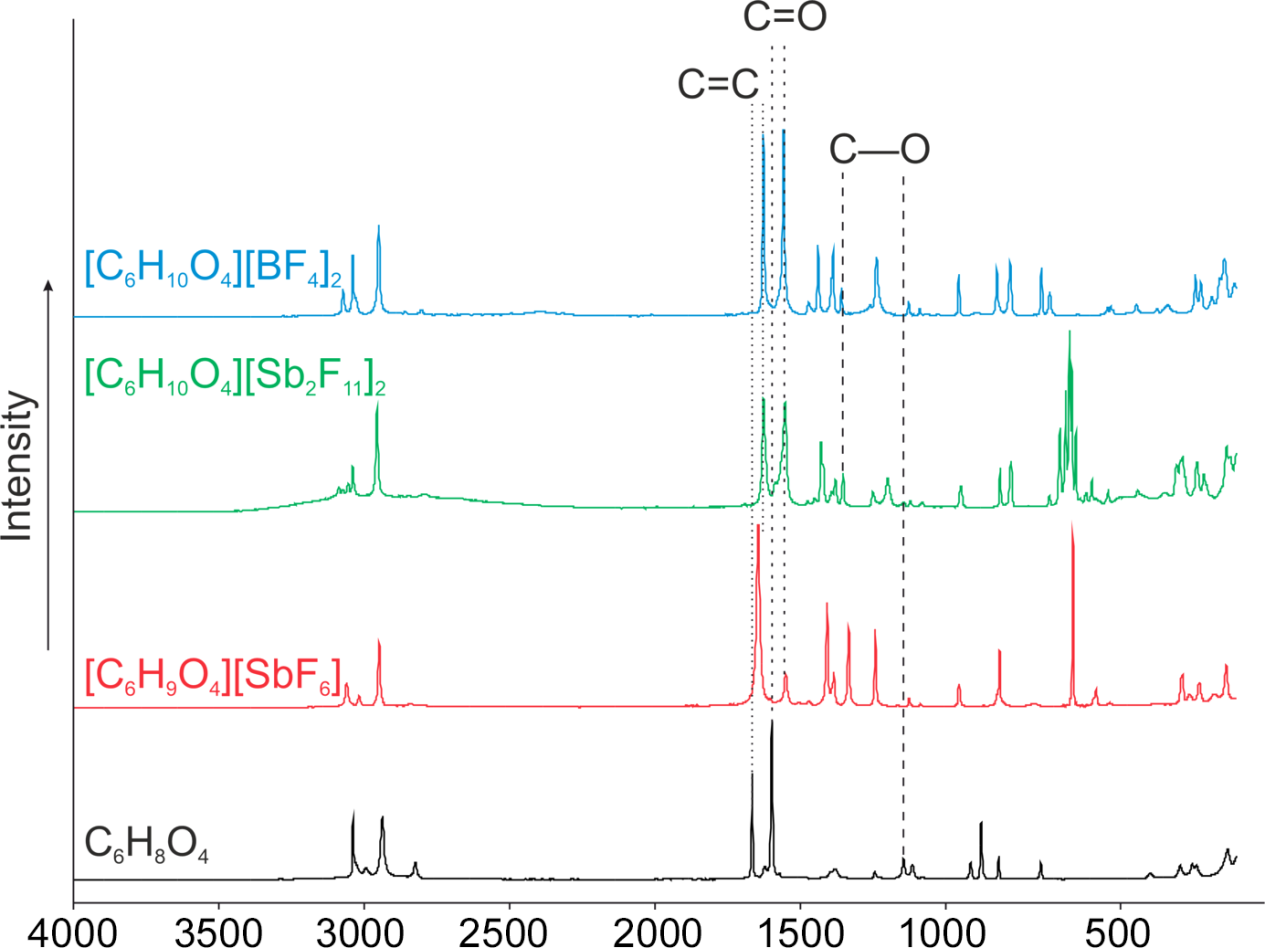
The low-tem­per­a­ture Raman spectra of **IV** (black), **V** (red), **VII** (green) and **VIII** (blue).

Fig. 15 ▸ shows the low-tem­per­a­ture Raman spectra of **IV**, **V**, **VII** and **VIII**. The C=O oscillation is red-shifted by 48 cm^−1^ to 1611 cm^−1^ com­pared to **IV** (1659 cm^−1^). Due to the pro­ton­ation, the C—O oscillation is blue-shifted in the Raman spectra to 1401 cm^−1^ (**V**) com­pared to the neutral com­pound (1217 cm^−1^). The C=C oscillation is only slightly red shifted to 1705 cm^−1^ com­pared to the starting material (1725 cm^−1^).

The diprotonation is characterized by a red-shifted C=O oscillation by 46 cm^−1^ to 1613 cm^−1^ in the spectra of **VII** and **VIII** com­pared to the Raman spectrum of **IV** (1659 cm^−1^). The C=C oscillation is significantly red-shifted by 38 cm^−1^ to 1686 cm^−1^ in the spectra of **VII** and **VIII** com­pared to the Raman spectrum of **IV** (1724 cm^−1^). Due to the protonation, the C—O oscillation is blue-shifted in the Raman spectra to 1419 (in **VII**) and 1425 cm^−1^ (in **VIII**) com­pared to the neutral com­pound (1217 cm^−1^).

## Conclusion

We present herein the first single-crystal X-ray diffraction and Raman spectroscopy study of the monoprotonated species of methyl prop-2-ynoate and mono- and diprotonated species of dimethyl fumarate. All three protonated species crystallize in the more stable *syn*–*anti* conformation. Furthermore, the first single-crystal structure of methyl prop-2-ynoate is reported. The protonated species are important inter­mediates of acid-catalyzed reactions.

## Supplementary Material

Crystal structure: contains datablock(s) I, III, IV, VI, VII, global. DOI: 10.1107/S2053229624011653/oj3025sup1.cif

Structure factors: contains datablock(s) I. DOI: 10.1107/S2053229624011653/oj3025Isup2.hkl

Structure factors: contains datablock(s) III. DOI: 10.1107/S2053229624011653/oj3025IIIsup3.hkl

Structure factors: contains datablock(s) IV. DOI: 10.1107/S2053229624011653/oj3025IVsup4.hkl

Structure factors: contains datablock(s) VI. DOI: 10.1107/S2053229624011653/oj3025VIsup5.hkl

Structure factors: contains datablock(s) VII. DOI: 10.1107/S2053229624011653/oj3025VIIsup6.hkl

CCDC references: 2407012, 2407013, 2407014, 2407015, 2407016

## Figures and Tables

**Figure 2:**
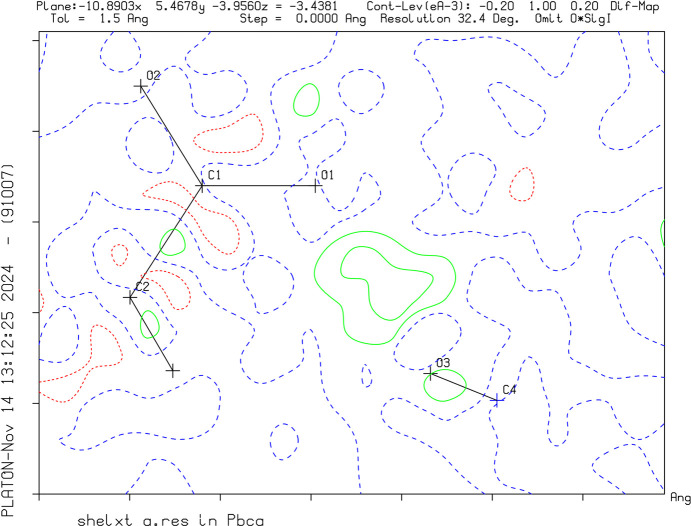
A difference Fourier map of **VI** without the H atom between O1 and F1. The green solid lines and red dotted lines show positive and negative density distribution, respectively.

**Figure 3:**
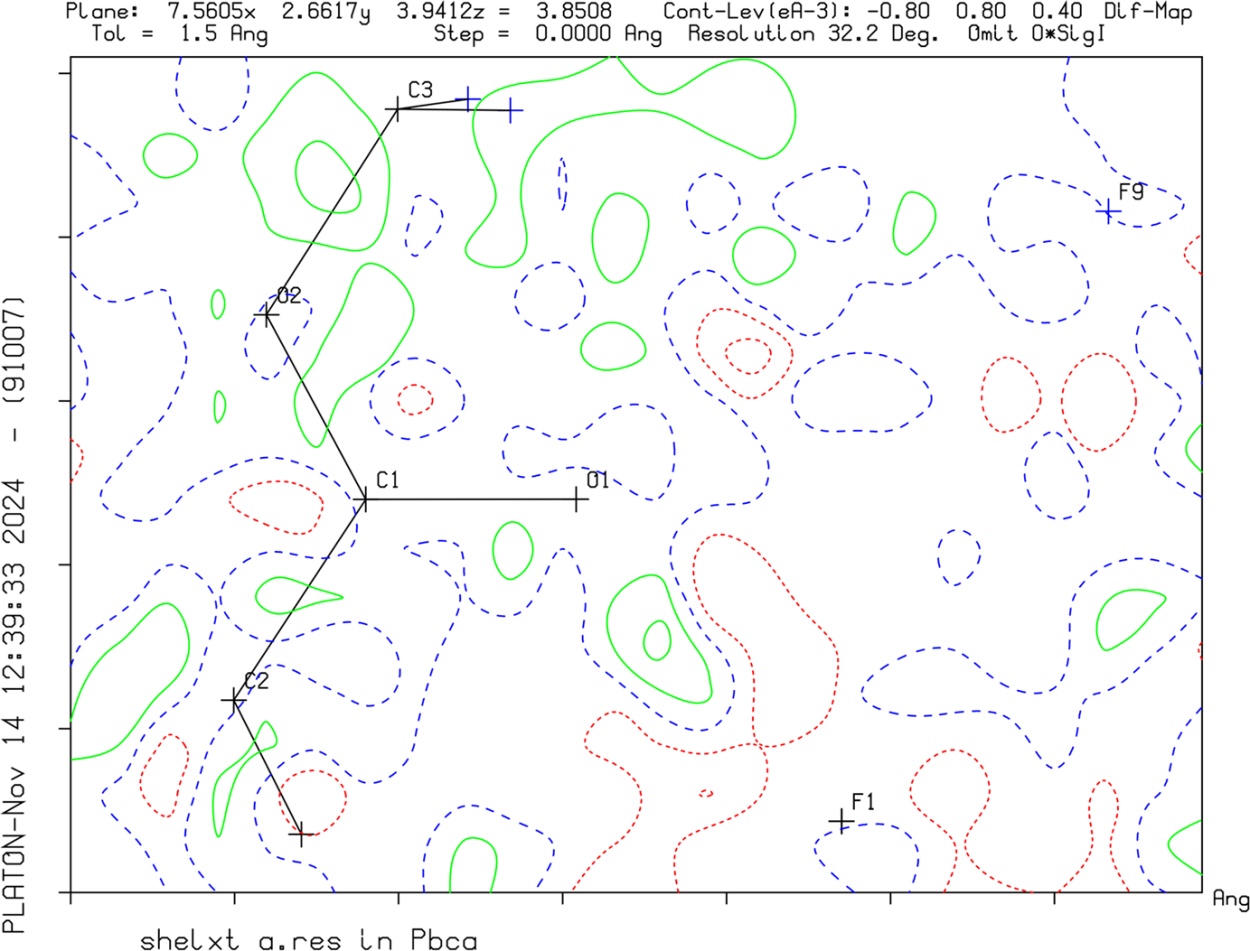
A difference Fourier map of **VII** without the H atom between O1 and F1. The green solid lines and red dotted lines show positive and negative density distribution, respectively.

**Table:** Experiments were carried out with Mo *K*α radiation using a Rigaku Xcalibur Sapphire3 diffractometer. Absorption was corrected for by multi-scan methods (*CrysAlis PRO*; Rigaku OD, 2020 ▸).

	**I**	**III**	**IV**
Crystal data			
Chemical formula	C_4_H_4_O_2_	C_4_H_5_O_2_^+^·AsF_6_^−^	C_6_H_8_O_4_
*M*_r_	84.07	274.00	144.12
Crystal system, space group	Monoclinic, *P*2_1_/*n*	Monoclinic, *P*2_1_/*n*	Triclinic, *P* 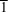
tem­per­a­ture (K)	111	111	112
*a*, *b*, *c* (Å)	3.8409 (5), 15.593 (2), 7.6149 (10)	6.9609 (5), 8.9319 (7), 13.7189 (9)	3.8726 (11), 5.6546 (10), 8.3778 (18)
α, β, γ (°)	90, 99.910 (12), 90	90, 91.664 (7), 90	100.642 (16), 100.42 (2), 105.73 (2)
*V* (Å^3^)	449.27 (10)	852.60 (11)	168.30 (7)
*Z*	4	4	1
μ (mm^−1^)	0.10	4.06	0.12
Crystal size (mm)	1.00 × 0.56 × 0.31	0.90 × 0.21 × 0.15	0.52 × 0.46 × 0.35

Data collection			
*T*_min_, *T*_max_	0.457, 1.000	0.582, 1.000	0.579, 1.000
No. of measured, independent and observed [*I* > 2σ(*I*)] reflections	3885, 1060, 891	4205, 2303, 1900	1403, 825, 703
*R*_int_	0.023	0.029	0.015
θ_max_ (°)	27.9	29.1	28.3
(sin θ/λ)_max_ (Å^−1^)	0.658	0.685	0.667

Refinement			
*R*[*F*^2^ > 2σ(*F*^2^)], *wR*(*F*^2^), *S*	0.036, 0.099, 1.05	0.039, 0.099, 1.05	0.048, 0.144, 1.04
No. of reflections	1060	2303	825
No. of parameters	71	123	51
No. of restraints	0	0	0
H-atom treatment	All H-atom parameters refined	H atoms treated by a mixture of independent and constrained refinement	H atoms treated by a mixture of independent and constrained refinement
Δρ_max_, Δρ_min_ (e Å^−3^)	0.18, −0.15	0.87, −0.92	0.52, −0.24

**Table:** 

	**VI**	**VII**
Crystal data		
Chemical formula	C_6_H_9_O_4_^+^·BF_4_^−^·2HF	0.5C_6_H_10_O_4_^2+^·Sb_2_F_11_^−^
*M*_r_	271.96	525.57
Crystal system, space group	Orthorhombic, *Pbca*	Orthorhombic, *Pbca*
tem­per­a­ture (K)	101	101
*a*, *b*, *c* (Å)	12.8759 (5), 11.8899 (4), 14.6252 (7)	7.8461 (6), 15.1531 (11), 19.5536 (17)
α, β, γ (°)	90, 90, 90	90, 90, 90
*V* (Å^3^)	2239.02 (16)	2324.8 (3)
*Z*	8	8
μ (mm^−1^)	0.19	4.79
Crystal size (mm)	0.28 × 0.18 × 0.11	0.25 × 0.11 × 0.05

Data collection		
*T*_min_, *T*_max_	0.885, 1.000	0.807, 1.000
No. of measured, independent and observed [*I* > 2σ(*I*)] reflections	7514, 1717, 1478	13316, 3784, 2878
*R*_int_	0.026	0.053
θ_max_ (°)	23.8	32.2
(sin θ/λ)_max_ (Å^−1^)	0.568	0.750

Refinement		
*R*[*F*^2^ > 2σ(*F*^2^)], *wR*(*F*^2^), *S*	0.040, 0.100, 1.05	0.036, 0.071, 1.04
No. of reflections	1717	3784
No. of parameters	176	169
No. of restraints	1	0
H-atom treatment	H atoms treated by a mixture of independent and constrained refinement	H atoms treated by a mixture of independent and constrained refinement
Δρ_max_, Δρ_min_ (e Å^−3^)	0.77, −0.35	1.09, −1.01

com­puter programs: *CrysAlis PRO* (Rigaku OD, 2020 ▸), *SHELXT* (Sheldrick, 2015*a* ▸), *SHELXL2018* (Sheldrick, 2015*b* ▸), *ORTEP-3 for Windows* (Farrugia, 2012 ▸) and *PLATON* (Spek, 2020 ▸).
